# Analytical Solution and Analysis of Aerodynamic Noise Induced by the Turbulent Flow Interaction of a Plate with Double-Wavelength Bionic Serration Leading Edges

**DOI:** 10.3390/biomimetics10040193

**Published:** 2025-03-21

**Authors:** Chenye Tian, Xiaomin Liu, Lei Wang, Yuefei Li, Yandong Wu

**Affiliations:** 1 School of Energy and Power Engineering, Xi’an Jiaotong University, Xi’an 710049, China; tianchenye@stu.xjtu.edu.cn (C.T.); 18821639689@163.com (L.W.); 2 Fudi Technology Research Institute, BYD Automotive Industry Co., Ltd., Shenzhen 518118, China; 3 Guangdong Midea Heating & Ventilating Equipment Co., Ltd., Foshan 528311, China; liyuef@midea.com (Y.L.); wuyandong@midea.com (Y.W.)

**Keywords:** aerodynamic noise, leading-edge serrations, optimized design of serrated structures, analytical solution, flow control, noise reduction mechanism

## Abstract

As a bionic flow control structure, leading-edge serrations have been proven to effectively suppress the aerodynamic noise of airfoils. Compared with single-wavelength serrations, a greater noise reduction potential can be obtained for airfoils with the double-wavelength serrations because of the phase interference at different tip-to-root ratios. In this study, in order to reduce the aerodynamic noise of a flat plate operating in a steady uniform flow, double-wavelength leading-edge serrations based on Ayton’s analytical model are optimized by the meta-heuristic optimization algorithm. The effects of different double-wavelength serrations on the noise characteristics of the flat plate are investigated. By comparing and analyzing the radiation integral function and quantifying the sound pressure along the leading edge of the flat plate, the local source cut-off effect resulting from the large transition curvature of the root and phase difference superposition is analyzed in detail. The results show that, before the first inflection point, the convex sinusoidal and iron-shaped serrations can significantly reduce the aerodynamic noise of the flat plate. When the concave ogee-shaped serrations are adopted, the reduction of the high-frequency noise is more obviously. Especially when the slits are embedded at the roots of the optimized leading-edge serrated structures, the improved design further promotes an additional noise reduction level of 0.7 dB for the flat plate. Through numerical studies, the coupled noise reduction mechanism of the serration roots and the slits is also revealed.

## 1. Introduction

The airfoil is a fundamental element that constitutes the blades of a wind turbine. Considering the public acceptance of wind turbines and environmental viability, developing efficient and low-noise airfoils is crucial for effectively improving the performance and reducing the noise of wind turbines. As we know, the airfoil noise can be divided into two types: self-noise and inflow-turbulence noise [1,2]. Airfoil self-noise is generally the result of interactions between the airfoil and its boundary layer, and it arises from flow instability in the boundary layer. Inflow-turbulence noise is caused by the interaction between the airfoil and the incoming unsteady wind gusts. In general, the leading-edge noise generated by the free-flow turbulence is the same as the trailing-edge noise, but the free-flow scale of the leading-edge boundary layer is larger than that of the trailing-edge turbulence. Therefore, the lower the leading-edge noise frequency, the larger its coherence scale and the stronger the noise radiation. Because of the interaction between complex atmospheric inflow and wind turbine blades [3], low-frequency broadband noise is generated.

Interest in developing methods and strategies to suppress the aerodynamic noise generation of airfoils is still growing. The noise-reducing effectiveness of the bionic leading-edge serrations has been scientifically confirmed by several researchers. However, most of these studies have focused on traditional triangular serrations [4,5,6,7]. On the other hand, although considerable achievements have been made in previous studies on the serrations, the experimental measurements and numerical simulations of the noise reduction mechanisms of bionic serrations for the airfoils still require significant time and material costs.

It is worth noting that the proposed analytical solution has important practical significance for highlighting the more essential noise reduction mechanism of leading-edge serrations for the flat plate. The most common approach to examining these noise reduction mechanisms is the study of the acoustic response between convective gusts and semi-infinite plates with serrations [8]. Sears carried out such experiments to establish the aerodynamic response of a two-dimensional flat plate subjected to sinusoidal gusts [9]. The theories of Kirchhoff and Curle are employed in Amiet’s approach [10] to obtain the far-field acoustic power spectrum of a single sinusoidal incoming gust in a subsonic flow. In principle, if the wavenumber spectrum of the vertical velocity fluctuations is accurately modeled, the far-field acoustic response can be robustly predicted. The analysis can be simplified by considering a particular wavenumber component of the turbulence received by the observer, and the results of the simplification show good agreement with experiments.

Amiet’s theoretical model is extended by Lyu et al. [11] and applied to the airfoils with leading-edge serrations. Fourier-series expansion and the Schwarzschild technique are employed to solve this model. The far-field acoustic power density is also established according to the incident turbulent velocity statistics. An analytical solution of the theoretical model proposed by Huang et al. [12] is closed by Fourier-series expansion and the Wiener–Hopf method. However, these theoretical analyses usually depend on standard Fourier-series expansions in the extended coordinates of the leading-edge serrations. Therefore, revealing the noise reduction mechanisms is rather more difficult. Envia [13] used the Fourier transform and the Wiener–Hopf method to calculate an approximate solution. This is conducive to obtaining expressions for the far-field acoustics and conducting a parametric study. Enlighted by this processing method, Ayton and Kim [14] proposed a more concise form of the analytical solution. Their method avoids the numerical step of solving the far-field acoustic response by converting the control equations and boundary conditions based on the variable transformation. This method provides theoretical support for rapidly evaluating the noise-reducing effectiveness of serrations.

Chaitanya et al. [15] proposed a double-wavelength serrated structure formed by the superposition of serrations of two different frequencies, amplitudes, and phases, and they evaluated its aerodynamic performance. They revealed the noise reduction mechanism in relation to source control and radiation control, and they concluded that double-wavelength serrations are more effective at reducing broadband noise than single-wavelength serrations. After applying the conclusions that they obtained using a flat plate to a 3D airfoil, they found that the overall sound suppression is almost equally effective, although the effect is weakened at the roots of the serrations. Recently, a new serration type with a parameterized profile was proposed by Lyu et al. [16], and it was evaluated using an analytical model based on the dimensionless streamwise hydrodynamic wavenumber. Compared with traditional serrations, the acoustic performance was improved within a specific frequency range, while the undesired deterioration was found to occur at low frequencies. Additionally, the correctness of the trend predicted by the analytical model was also verified by experiments. In another study, leading-edge serrations possessing multiple bionic elements were embedded into an airfoil [17]. In comparison with traditional serrations, an additional noise attenuation level of 3.4 dB was achieved by the inclusion of iron-shaped serrations. In conclusion, serrations have potential for suppressing the aerodynamic noise of airfoils [18], and greater noise reduction levels can be achieved by optimizing the serrated structures.

For the wings of natural birds, the wavelengths of the non-smooth-edge serrations are non-uniformly distributed. Therefore, the noise reduction effect of serrations comprised of two frequency components of different amplitudes and phases is worth exploring. The phase interference principle and noise reduction mechanisms involved are critical for further sound suppression. The slits are also considered at the root of serrations for further noise reduction. Moreover, the aerodynamic performance of airfoils is inevitably affected by the serrated structures. Based on relevant experimental results [19], leading-edge serrations have a relatively small impact on the aerodynamic performance of symmetric airfoils. At a small angle of attack, the aerodynamic performance of an airfoil is even improved by leading-edge serrations. However, the negative impact of serrations on the aerodynamic performance of asymmetric airfoils still exists. Therefore, the laws of the influence of serrations on aerodynamic performance are complex. Generally, simple estimations are conducted using previous experimental experience or computational fluid dynamics. Combining a study [18] and relative engineering experience, amplitudes have a higher sensitivity to aerodynamic performance. Therefore, the amplitude of the serrations is determined through empirical coefficients at first, and then the shape of serrations is selected.

Specific serrations exhibit different acoustic performances with varying tip-to-root ratios. For this reason, it is difficult to establish a serrated structure that will be the most appropriate for a given application from a single experiment or numerical simulation. The advantage of an analytical solution is that if the scattered sound fields from any leading-edge serrations could be determined, the acoustic responses under the particular flow parameters would be rapidly predicted. As a result, many repeated experimental designs and a large amount of time-consuming numerical simulations are avoided. In our previous work [20], the noise-reducing abilities of leading-edge serrations of different shapes with different tip-to-root ratios for a flat plate were examined, based on the theory of Ayton and Chaitanya [8]. However, the orthogonal experimental method was used in the previous work, and the optimal design of the leading-edge serrations was limited by the selected range of the parameters. In this study, a meta-heuristic optimization algorithm is introduced into the analytical solution to seek and assess optimal double-wavelength serration profiles at specific tip-to-root ratios. The weights of the salient influences are further analyzed for different amplitudes, which is important for obtaining performance gains. Through a numerical analysis, the understanding of the essential noise-mitigating mechanisms is deepened. To improve the accuracy of the predictive model in the high-frequency range, a trailing-edge self-noise model is also considered. This study will provide a valuable theoretical reference for the noise reduction design of airfoils.

## 2. Analytical Formulation

The noise reduction mechanism of double-wavelength structures is investigated by combining the analytical model proposed by Ayton and Chaitanya [8] and a trailing-edge self-noise model. The corresponding theory provides the fast-response approach to acoustic prediction, and its effectiveness has been verified by corresponding experimental and numerical results [16,21]. A low-noise leading-edge shape can be quickly obtained after determining the flow parameters of the leading-edge geometry.

**Figure 1:**
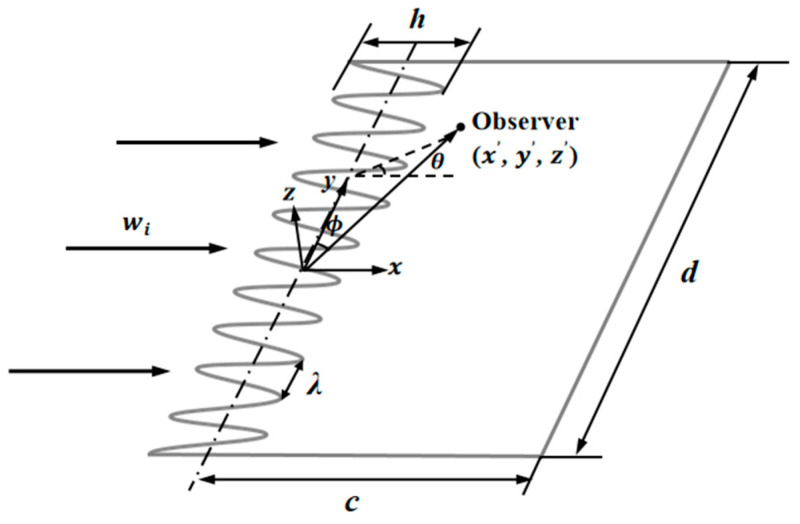
Sketch and coordinate system of the leading edge.

The model of these serrations is simplified, as shown in Figure 1. Here, a zero-thickness semi-infinite plate at a 0° attack angle placed in a mean incoming component of an incident gust is supposed to represent a serrated airfoil; *x*, *y*, and *z* denote the streamwise direction, the spanwise direction, and the wall-normal direction, respectively [22]. Furthermore, Taylor’s frozen hypothesis is applied in the gusts that are assumed to be a two-dimensional spectrum. Significantly, the airfoil and serrations are periodic in the spanwise direction. The span length is much more than the spanwise correlation length of the boundary-layer turbulence [23].

In the model, all boundaries are away from any discontinuities of the leading edge. The settings are adopted to ensure that the influence of a discontinuous function can be avoided. The shape function F(y) is restricted to a single-value piecewise linear function, and quasi-periodic boundary conditions are imposed at 0 and 1. Therefore, the spanwise region of the unit length is limited in the range of 0≤y≤1. The tip-to-root length, which is related to the sharpness of the serrations, is defined as h, and the geometric functions of the serrations are modeled as $0.5h\cdot F\left(y\right)$. Additionally, $\bar{h}$ represents the dimensionless tip-to-root length that is normalized by the wavelength λ.

An analytical solution for the acoustic results at a given frequency is obtained using Wiener–Hopf equation method and Fourier transform to separate variables from the convection Helmholtz equation. A more detailed process can be derived from reference [22]. The power spectral density is obtained by the following equation:

(1) $$\psi =\frac{1}{\pi }\cos^{2}\frac{\theta }{2}\int_{-\infty }^{\infty }{\left|\sum_{n=-\infty }^{\infty }\frac{k_{1}/\beta^{2}-w_{n}\cos\theta }{{\bar{k}}_{1}-w_{n}\cos\theta }\frac{1}{\sqrt{{\bar{k}}_{1}+w_{n}}}\frac{e^{iw_{n}r}}{\sqrt{r}}e^{i\chi_{n}y}E_{n}\left(-w_{n}\cos\theta \right)\right|}^{2}\Phi^{\left(\infty \right)}\left(k_{1},k_{2}\right)dk_{2}$$

where θ is far-field monitoring point. $k_{1}=k/M$, where *M* is defined as $M=U/c_{0}$ and $c_{0}$ is the speed of sound. $\beta^{2}=1-M^{2}$; ${\bar{k}}_{1}=k_{1}/c_{0}$; $w_{n}^{2}={\left(\delta M\right)}^{2}-\chi_{n}^{2}$, $\chi_{n}=\pm k_{2}+2n\pi$, $\delta =k_{1}/\beta$. The radiation integral function $E_{n}$ is defined by

(2) $$E_{n}(-w_{n}\cos\theta )=\int_{0}^{1}e^{i({\bar{k}}_{1}-w_{n}\cos\theta )\bar{\Theta }F\left(\eta \right)}e^{-i2n\pi \eta }d\eta ,$$

where $\bar{\Theta }=h/\beta$.

The energy spectrum (Liepmann spectrum) of the upstream vertical fluctuation is defined by

(3) $$\Phi^{\left(\infty \right)}\left(k_{1},k_{2}\right)=\frac{3{u^{\prime }}^{2}\Lambda^{2}}{4\pi }\frac{\Lambda^{2}\left(k_{1}^{2}+k_{2}^{2}\right)}{{\left[1+\Lambda^{2}\left(k_{1}^{2}+k_{2}^{2}\right)\right]}^{5/2}}$$

where Λ is the integral length scale of turbulence. The integral scale estimated by the turbulence spectrum measured by hot wire anemometry is set as 7.5 mm. $u^{\prime }$ represents the turbulent intensity, which is defined as 2.5% of the free-stream velocity. The free-stream velocity is set as 60 m/s.

**Figure 2:**
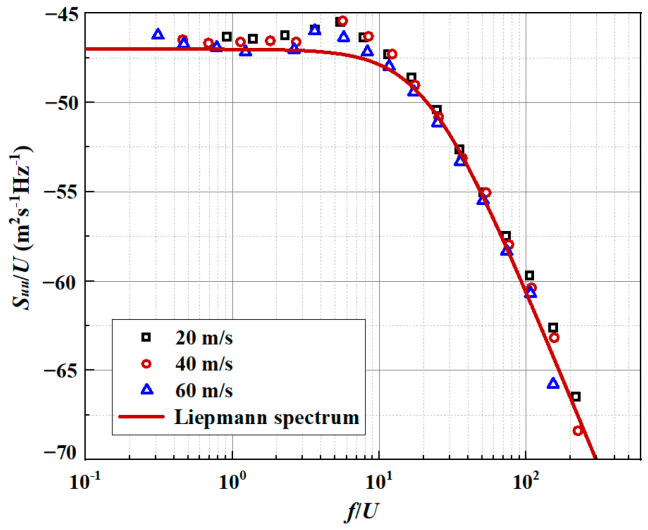
Comparison between the measured streamwise velocity energy spectra and theoretical analytical spectra.

Consistent with our previous work [20], the experimental data of the mean velocity fluctuation spectrum for the incoming airflow velocities of 20 m/s, 40 m/s, and 60 m/s are also extracted in this paper. A sampling frequency of 50 KHz and a window size with 1024 data points are used in this analytical model solution. More details on the implementation of the experiment can be found reference [24]. As depicted in Figure 2, the energy spectra of different velocity fluctuations can be well reflected above both analytical energy spectra. It can be shown that the distribution of the theoretical Liepmann spectra is in good agreement with the experimental data over the overall frequency range. The homogeneous turbulence spectrum is estimated by Taylor’s hypothesis [25]. Notably, division by the mean velocity is necessary to ensure that the energy spectrum integrates with the mean square velocity fluctuation.

## 3. Experimental Verification

A flat plate is established to complete the fundamental parameter settings in this study. The plate is set with a mean chord of 150 mm, and its span is set to three times the chord. The emission angle range of the microphone receiving the sound source is set to 40–140°. The receiving points are located on the arc with a radius of 1.2 m from the leading edge of the flat plate. The serrations with tip-to-root ratios $\bar{h}$ of 0.5, 1.0, and 2.0 are implemented on the plate to analyze the acoustic sensitivity of the sound source. As the tip-to-root ratio increases, the analytical results of Lyu’s model [22] are found to differ greatly from the experimental results over the high-frequency band without considering the trailing-edge self-noise. Generally, the chord length of the investigated airfoil is finite, and so trailing-edge self-noise will inevitably be generated by the experimental device. That is to say, the effectiveness of the noise reduction resulting from the leading-edge serrations will be limited by the emission of trailing-edge self-noise, as noted in Narayanan’s 2018 work [24]. Therefore, trailing-edge self-noise is taken into account during the acoustic evaluation conducted in this study.

The generation of trailing-edge self-noise originates from the interaction between solids and flow turbulence. This interaction generates pressure fluctuations, leading to energy scattering and converting the turbulent kinetic energy into sound energy [26]. It is essentially broadband noise [27]. Brooks et al. [28] elucidate five self-noise mechanisms under subsonic flow conditions. Based on the edge scattering formula proposed by Ffowcs Williams and Hall, the trailing-edge noise of the turbulent boundary layer can be solved with the average turbulent boundary layer thickness as the required length scale. Brooks and Marcolini [29] conducted experimental research to quantitatively separate the tip noise. A quantitative prediction method has been proposed from the perspective of data scaling. Grosveld’s study [30] found that scaling models can explain the spectral behavior of the broadband noise at high frequency in wind turbines. Chou and George [31] constructed corresponding noise-solving models for previous noisy data by replacing scaling. But, they did not consider the variations in the boundary layer thickness and specific shape details. Therefore, a more universal and accurate model becomes particularly important.

The BPM method for predicting trailing-edge self-noise was proposed by Brooks, Pope, and Marcolini in 1989 [27], which established the self-noise mechanisms caused by five particular boundary layers. In their work, a series of aeroacoustic results based on airfoil sections were verified in an anechoic wind tunnel, along with the broadband noise from a large helicopter rotor model. Since the BPM method has the advantage of combining a large number of empirical parameters related to experiments, it has become an important method for some complex noise prediction schemes.

The overall sound pressure level generated by the turbulent boundary layer trailing edge is given by

(4) $${\mathrm{SPL}}_{\mathrm{TE}}=10\log\left(10^{{\mathrm{SPL}}_{\alpha }/10}+10^{{\mathrm{SPL}}_{s}/10}+10^{{\mathrm{SPL}}_{p}/10}\right)$$

where

(5) $${\mathrm{SPL}}_{p}=10\log\left(\frac{\delta_{p}^{*}M^{5}L{\bar{D}}_{h}}{r_{e}^{2}}\right)+A\left(\frac{St_{p}}{St_{1}}\right)+\left(K_{1}-3\right)+\Delta K_{1}$$

(6) $${\mathrm{SPL}}_{s}=10\log\left(\frac{\delta_{s}^{*}M^{5}L{\bar{D}}_{h}}{r_{e}^{2}}\right)+A\left(\frac{St_{s}}{St_{1}}\right)+\left(K_{1}-3\right)$$

(7) $${\mathrm{SPL}}_{\alpha }=10\log\left(\frac{\delta_{\alpha }^{*}M^{5}L{\bar{D}}_{h}}{r_{e}^{2}}\right)+B\left(\frac{St_{\alpha }}{St_{2}}\right)+K_{2}$$

(8) $$St_{p}=\frac{f\delta_{p}^{*}}{U},St_{s}=\frac{f\delta_{s}^{*}}{U}$$

(9) $$St_{1}=0.02M^{-0.6}$$

(10) $$St_{2}=St_{1}\times \left\{\begin{array}{l} 1,\left(\alpha_{*}<1.33^{\circ }\right)\\ 10^{0.0054{\left(\alpha_{*}-1.33\right)}^{2}},\left(1.33^{\circ }<\alpha_{*}<12.5^{\circ }\right)\\ 4.72,\left(\alpha_{*}>12.5^{\circ }\right) \end{array}\right.$$

(11) K1=−9.0logRe+181.6,2.47×105≤Rc≤8×105

(12) $$\Delta K_{1}=0,\left(R>5000\right)$$

(13) $${\bar{D}}_{h}\left(\theta_{e},\Phi_{e}\right)\approx \frac{2\sin^{2}\left(\theta_{e}/2\right)\sin^{2}\Phi_{e}}{\left(1+M\cos\theta_{e}\right)\left[1+\left(M-M_{c}\right)\cos\theta_{e}\right]}$$

Here, ${SPL}_{p}$ and $\delta_{p}^{*}$ denote the sound pressure levels and the boundary-layer displacement thicknesses caused by the pressure side, respectively; ${SPL}_{s}$ and $\delta_{s}^{*}$ denote the sound pressure levels and the boundary-layer displacement thicknesses caused by the suction side, respectively; ${SPL}_{\alpha }$ and $\delta_{\alpha }^{*}$ denote the angle-dependent sound pressure level and the boundary-layer displacement thicknesses caused by the angle of attack, respectively; *L* is the span length; ${\bar{D}}_{h}$ is the directivity function; and Θ is the angle from the source streamwise axis *x* to the observer. Φ is the angle from the source lateral axis *y* to the observer. The subscript *e* indicates the retarded coordinate. The overbar of ${\bar{D}}_{h}$ denotes that it is normalized by the trailing-edge noise radiated in the direction of $\Theta_{e}=90^{{}^{\circ}}$ and $\Phi_{e}=90^{{}^{\circ}}$. Therefore, ${\bar{D}}_{h}(90^{{}^{\circ}},90^{{}^{\circ}})$ = 1. More details with respect to definitions can be obtained from Appendix B in reference [27]. The coefficient *A* is the spectral shape function for the trailing-edge noise caused by the turbulent boundary layer, and *B* is the separation noise. *K*_1_ and $\Delta K_{1}$ are constants; $R_{S_{p}}^{*}$ is the Reynolds number based on pressure-side displacement thickness. ${St}_{p}$, ${St}_{s}$, ${St}_{1}$, and ${St}_{2}$ denote the different Strouhal numbers associated with the turbulent boundary layer trailing edge and separation noise scaling, respectively. $r_{e}$ represents the distance between the receiving point of the sound source and the trailing edge ($r_{e}=1.22\;m$).

**Figure 3:**
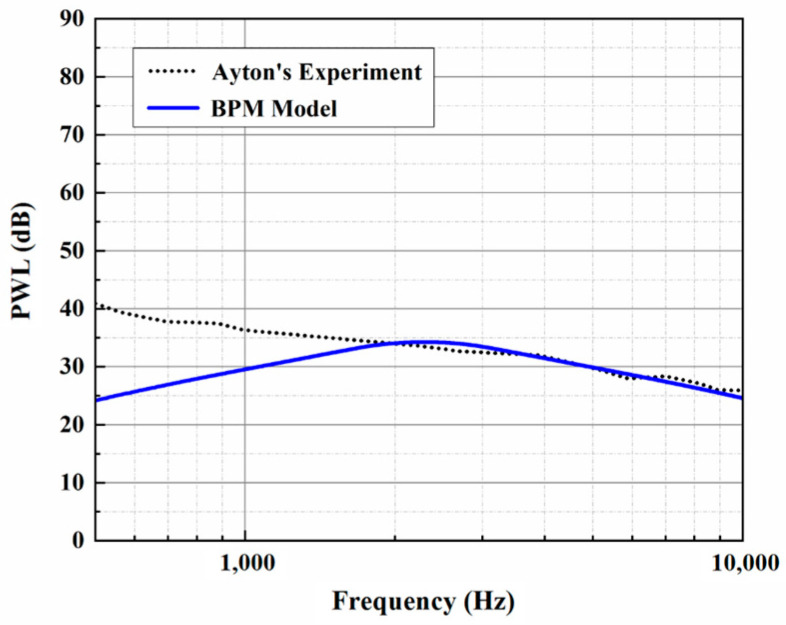
Comparison of trailing-edge self-noise based on the analytical solution and experimental results.

**Figure 4:**
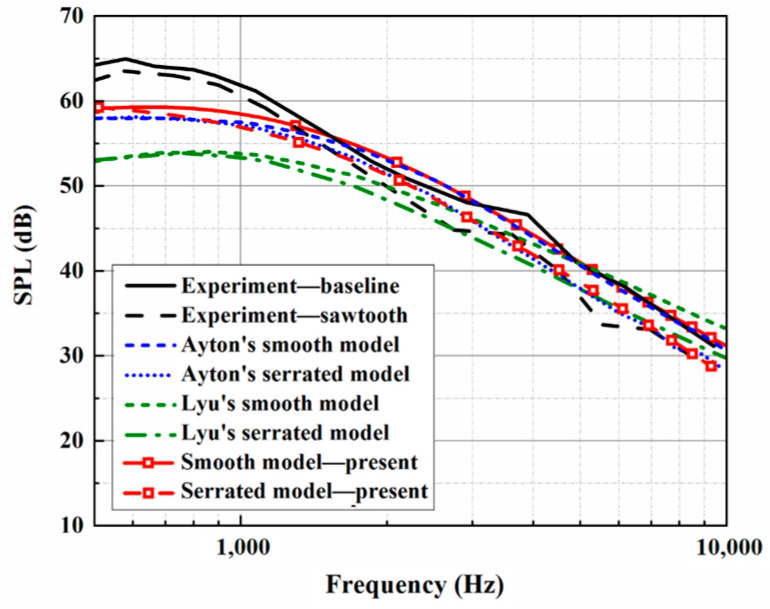
Sound pressure level spectra considering self-noise alongside the experimental results at a tip-to-root ratio of 0.5.

**Figure 5:**
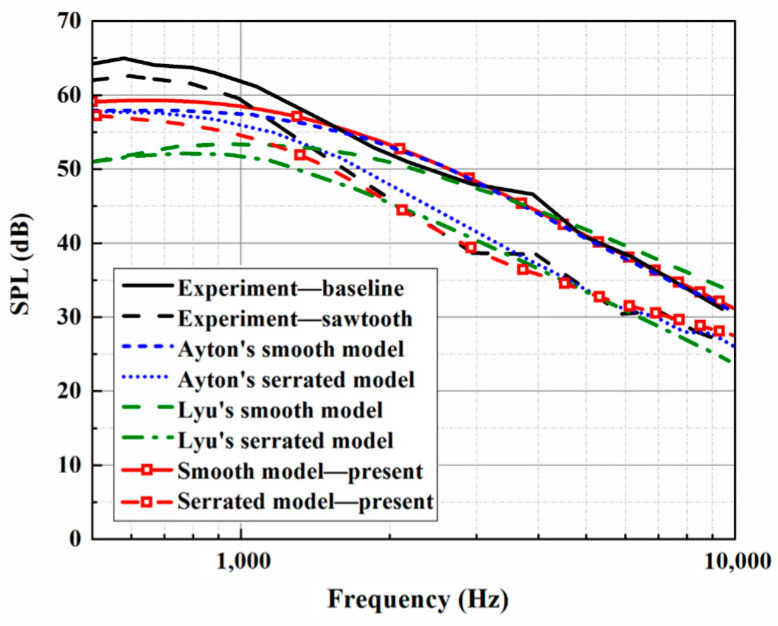
Sound pressure level spectra considering self-noise alongside the experimental results at a tip-to-root ratio of 1.

**Figure 6:**
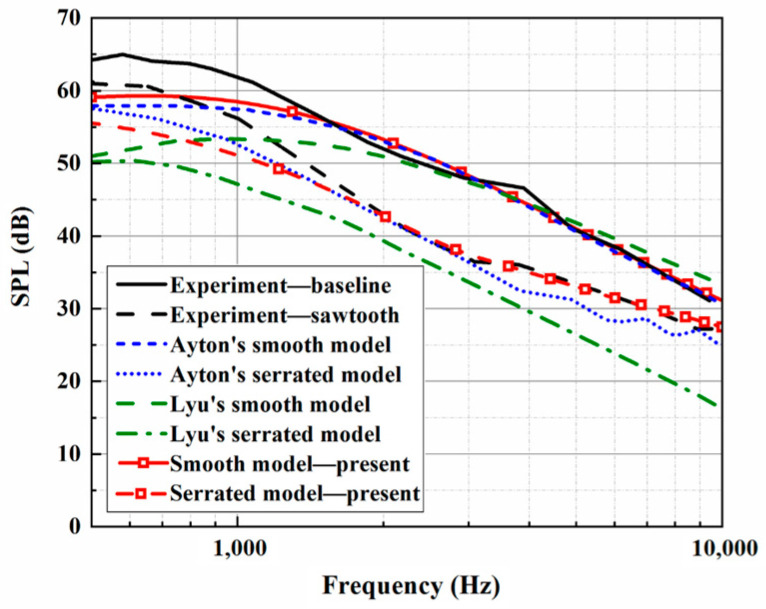
Sound pressure level spectra considering self-noise alongside the experimental results at a tip-to-root ratio of 2.

The trailing-edge parameters in this study are selected and used for the analytical sound prediction, which coincide with the corresponding experimental data for f>2000 Hz, as shown in Figure 3. Based on that, the effectiveness of the BPM method is considered to have been verified. It is worth noting that trailing-edge self-noise plays an important role in an acoustic evaluation at larger $\bar{h}$ values. A larger *h* promotes a reduction in LE noise, making self-noise function over a wider frequency range. When considering trailing-edge self-noise, the sound pressure level distributions show good agreement between Ayton’s model and the experimental data in the high-frequency region, as shown in Figure 4, Figure 5 and Figure 6. Moreover, it is observed that the BPM method is highly accurate at predicting the self-noise acoustic spectra.

However, some deviation between the analytical sound prediction and the experimental data can also be observed at lower frequencies. Based on a previous study [8], when at low frequencies, jet noise from the nozzle dominates the experimental measurements, and typical jet shear-layer noise is also present in the self-noise spectra. The offset between the analytical models and the experiment at low frequencies is mainly dominated by jet noise. On the other hand, the model used for the experiments is of finite size, but that used for the noise predictions is assumed to have a semi-infinite chord length. Therefore, the Kutta condition cannot be applied at the trailing edge in the noise predictions. The Kutta condition is an important reason for the generation of velocity circulation, thus affecting the trailing-edge noise by affecting the pressure distribution at the trailing edge. According to the study carried out by Amiet [10], the condition of $cf/(c_{0}\beta^{2})<\pi /4$ is important. It means that the Kutta condition in the low-frequency range will also have a certain impact. The analytical model does not apply the Kutta conditions, which can also lead to a discrepancy with the experimental results at lower frequencies.

## 4. Mathematical Modeling and Optimization of Double-Wavelength Serrations

**Figure 7:**
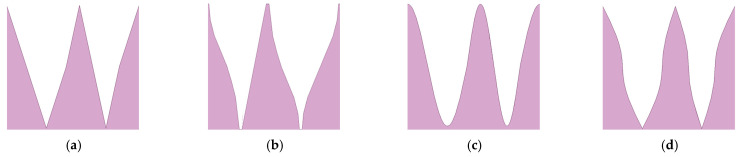
Four kinds of serrations with different curvatures applied to the leading edge of a flat plate. (**a**) Traditional serrations. (**b**) Ogee-shaped serrations. (**c**) Sinusoidal serrations. (**d**) Iron-shaped serrations.

Double-wavelength serrations based on phase interference provide a new concept for the noise reduction design of the airfoil. In our previous work [20], four different kinds of double-wavelength serrations were examined to systematically study the influence of their relevant parameters on reducing noise, as shown in Figure 7.

**Figure 8:**
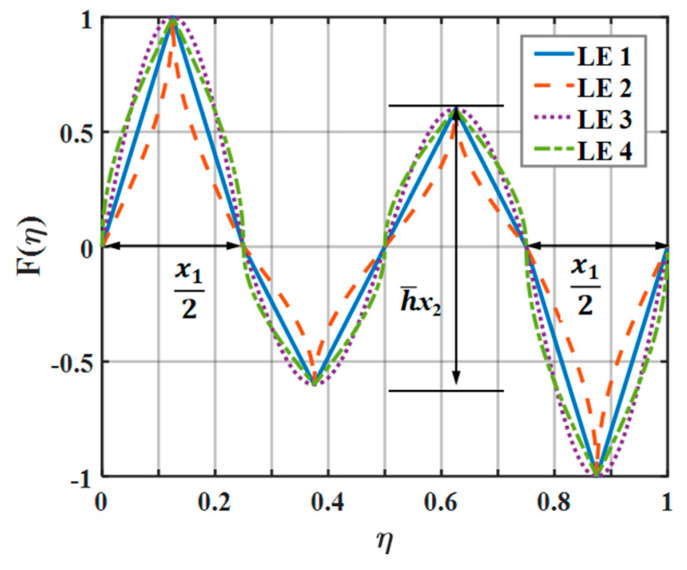
Schematic plot of a double-wavelength serration.

The parameter settings regarding double-wavelength serrations are described in Figure 8. It is seen that the coefficients of the wavelength and amplitude for the main serrations are $x_{1}$ and one, respectively. The coefficients of the wavelength and amplitude for the assisted serrations are 1 − *x*_1_ and *x*_2_, respectively. LE 1–LE 4 denote the traditional serrations, ogee-shaped serrations, sinusoidal serrations, and iron-shaped leading-edge serrations, respectively.

The multi-factor sensitivity analysis of double-wavelength serrations was also investigated in our previous work [20], including shape factors, wavelengths, and amplitudes. This informs the general optimization direction for the serrations. However, because of the orthogonal experimental method used in our previous work, the discovery of serrations with good noise reduction depends entirely on the number of selected test samples; so, the proposal of the optimal serrations still needs to rely on the optimization algorithm for a solution. In this study, a meta-heuristic optimization algorithm is employed to obtain the best noise-reducing effect of double-wavelength serrations with a particular tip-to-root ratio. The whale optimization algorithm is sufficiently competitive when compared to state-of-art meta-heuristic algorithms and other conventional methods [32,33]. Due to its adaptive spiral-updating mechanism, which balances exploration and exploitation more efficiently than the mutation/crossover operators of genetic algorithms or the velocity-based updates of particle swarm optimization, the whale optimization algorithm achieves faster convergence in high-dimensional optimization problems [32].

Based on this, in a specific operation condition, the minimization of the overall sound pressure level (OASPL) is regarded as the objective to achieve the purpose of reducing noise emissions. The aeroacoustic problem of a flat plate at a zero angle of attack can be described as follows:

Consider $\vec{x}=[x_{1}x_{2}]$.

Minimize f(x)=OASPL.

subject to

(14) g(x)≤0,i=1,⋯,m

with variable ranges $0\leq x_{1}\leq 1$,

$$0\leq x_{2}\leq 1$$

Here, $\vec{x}$ represents the vector of variables, and f(x) is the objective function. The constraints yield the aeroacoustic conditions. Equations (15)–(17) represent the different leading-edge shape functions.

(15) $$F\left(\eta \right)=\left\{\begin{array}{ll} \frac{\arcsin\left(m_{1}\eta \right)}{\arcsin\left(m_{1}/m_{3}\right)}, & 0\leq \eta \leq \frac{x_{1}}{4}\\ \frac{\arcsin\left(-m_{1}\left(\eta -x_{1}/2\right)\right)}{\arcsin\left(m_{1}/m_{3}\right)}, & \frac{x_{1}}{4}\leq \eta \leq \frac{x_{1}}{2}\\ \frac{\arcsin\left(-m_{2}\left(\eta -x_{1}/2\right)\right)}{\arcsin\left(m_{2}/m_{4}\right)}, & \frac{x_{1}}{2}\leq \eta \leq \frac{x_{1}}{2}+\frac{1-x_{1}}{4}\\ \frac{\arcsin\left(m_{2}\left(\eta -1/2\right)\right)}{\arcsin\left(m_{2}/m_{4}\right)}, & \frac{x_{1}}{2}+\frac{1-x_{1}}{4}\leq \eta \leq \frac{1}{2}+\frac{1-x_{1}}{4}\\ \frac{\arcsin\left(-m_{2}\left(\eta -\left(1-x_{1}/2\right)\right)\right)}{\arcsin\left(m_{2}/m_{4}\right)}, & \frac{1}{2}+\frac{1-x_{1}}{4}\leq \eta \leq 1-\frac{x_{1}}{2}\\ \frac{\arcsin\left(-m_{1}\left(\eta -\left(1-x_{1}/2\right)\right)\right)}{\arcsin\left(m_{1}/m_{3}\right)}, & 1-\frac{x_{1}}{2}\leq \eta \leq 1-\frac{x_{1}}{4}\\ \frac{\arcsin\left(m_{1}\left(\eta -1\right)\right)}{\arcsin\left(m_{1}/m_{3}\right)}, & 1-\frac{x_{1}}{4}\leq \eta \leq 1\\ m_{1}=\frac{m_{\circ }}{x_{1}},m_{1}=\frac{m_{\circ }}{1-x_{1}},m_{3}=\frac{4}{x_{1}},m_{4}=\frac{m_{\circ }}{x_{1}} & \end{array}\right.$$

(16) $$F\left(\eta \right)=\left\{\begin{array}{ll} \sin\left(\frac{2\pi }{x_{1}}\eta \right), & 0\leq \eta <\frac{x_{1}}{2}\\ \sin\left(\frac{2\pi }{1-x_{1}}\left(\eta -\frac{1}{2}\right)\right), & \frac{x_{1}}{2}\leq \eta \leq \frac{1}{2}+\frac{1-x_{1}}{2}\\ \sin\left(\frac{2\pi }{x_{1}}\left(\eta -1\right)\right), & \frac{1}{2}+\frac{1-x_{1}}{2}<\eta \leq 1 \end{array}\right.$$

(17) $$F\left(\eta \right)=\left\{\begin{array}{ll} \frac{\arcsin\left(m_{1}\left(\eta -x_{1}/4\right)\right)}{\arcsin\left(m_{1}/m_{3}\right)}+1, & 0\leq \eta <\frac{x_{1}}{4}\\ \frac{\arcsin\left(-m_{1}\left(\eta -x_{1}/4\right)\right)}{\arcsin\left(m_{1}/m_{3}\right)}+1, & \frac{x_{1}}{4}\leq \eta <\frac{x_{1}}{2}\\ \frac{\arcsin\left(-m_{2}\left(\eta -\left(x_{1}/2-\left(1-x_{1}\right)/4\right)\right)\right)}{\arcsin\left(m_{2}/m_{4}\right)}-1, & \frac{x_{1}}{2}\leq \eta <\frac{x_{1}}{2}+\frac{1-x_{1}}{4}\\ \frac{\arcsin\left(m_{2}\left(\eta -\left(x_{1}/2-\left(1-x_{1}\right)/4\right)\right)\right)}{\arcsin\left(m_{2}/m_{4}\right)}-1, & \frac{x_{1}}{2}+\frac{1-x_{1}}{4}\leq \eta <\frac{1}{2}\\ \frac{\arcsin\left(m_{2}\left(\eta -\left(1/2+\left(1-x_{1}\right)/4\right)\right)\right)}{\arcsin\left(m_{2}/m_{4}\right)}+1, & \frac{1}{2}\leq \eta <\frac{1}{2}+\frac{1-x_{1}}{4}\\ \frac{\arcsin\left(-m_{2}\left(\eta -\left(1/2+\left(1-x_{1}\right)/4\right)\right)\right)}{\arcsin\left(m_{1}/m_{3}\right)}, & \frac{1}{2}+\frac{1-x_{1}}{4}\leq \eta <\frac{1}{2}+\frac{1-x_{1}}{2}\\ \frac{\arcsin\left(-m_{1}\left(\eta -\left(1-x_{1}/4\right)\right)\right)}{\arcsin\left(m_{1}/m_{3}\right)}-1, & \frac{1}{2}+\frac{1-x_{1}}{2}\leq \eta <1-\frac{x_{1}}{4}\\ \frac{\arcsin\left(m_{1}\left(\eta -\left(1-x_{1}/4\right)\right)\right)}{\arcsin\left(m_{1}/m_{3}\right)}-1, & 1-\frac{x_{1}}{4}\leq \eta \leq 1 \end{array}\right.$$

The parameterized mathematical expressions for the standard serrations and the ogee serrations are modeled using Equation (15), which can be intuitively reflected in LE 1 and LE 2 in Figure 8, respectively. The sinusoidal and iron-shaped serrations are parameterized using the above Equations (16) and (17), which manifests in LE 3 and LE 4 in Figure 8, respectively. Here, *m* is the shape coefficient, which quantifies the sharpness of serrations and controls the curvature between every two inflection points of the shape curves. For standard serrations, *m* → 0. Additionally, *m* is set to four for the ogee serrations and the mentioned iron-shaped serrations. The length dimensions in the parameterized expressions below are normalized by the wavelength λ, and the wavelengths of the double wavelength serrations are set as *x*_1_ and 1 − *x*_1_.

**Figure 9:**
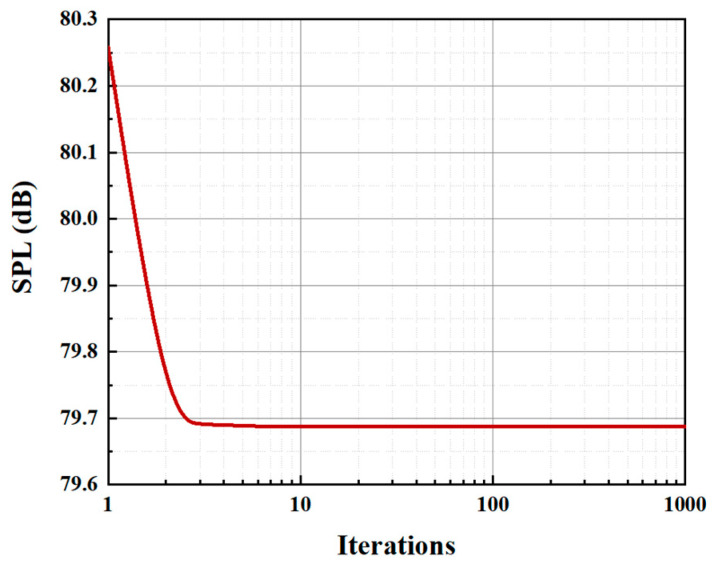
An example iterative convergence curve of the optimization.

In the data setup of the double-wavelength serrations, the amplitude coefficient *x*_1_ is always one, which is controlled by the dominant serrations, and the amplitude of the embedded serrations is represented by the amplitude coefficient *x*_1_. In limiting the parameters associated with the dominant wavelength, the dimensions of the optimized design space are reduced. The value ranges of $x_{1}$ and $x_{2}$ are defined as $\left[0,1\right]$. During the optimization process, the number of agent models is selected as 20, and the number of iteration steps is set to 1000. It can be seen from the representative convergence curve illustrated in Figure 9 that the optimal sound pressure level is stable.

**Figure 10:**
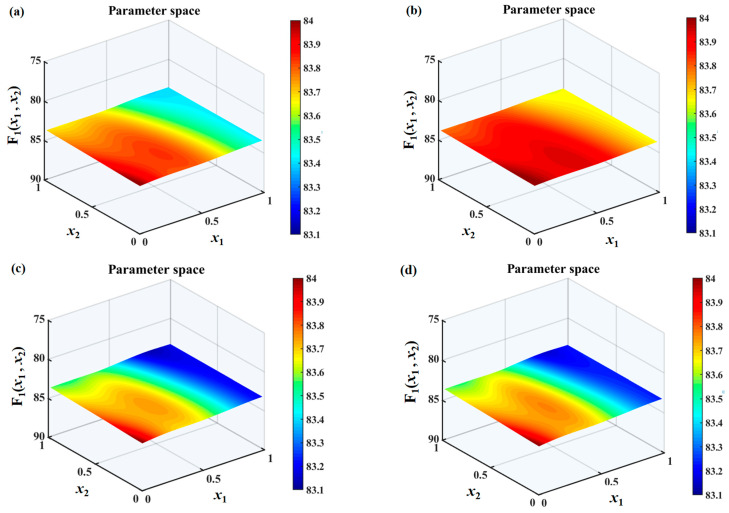
Sound pressure level distributions of a flat plate with double-wavelength serrations embedded and the optimization of the parameters of the wavelength coefficients (*x*_1_) of the main serrations and the amplitude coefficients (*x*_2_) of the assisted serrations in the search space at a frequency range of 0–10,000 Hz with a tip-to-root ratio of 0.5, including (**a**) traditional serrations, (**b**) ogee-shaped serrations, (**c**) sinusoidal serrations, and (**d**) iron-shaped serrations.

**Figure 11:**
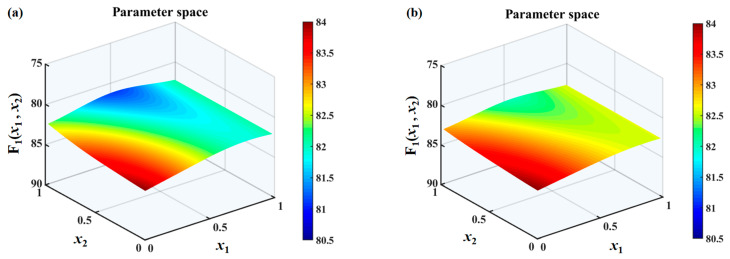

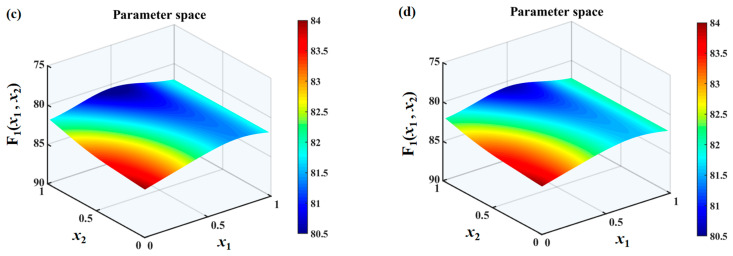
Sound pressure level distributions of a flat plate with double-wavelength serrations embedded and the optimization of the parameters of the wavelength coefficients (*x*_1_) of the main serrations and the amplitude coefficients (*x*_2_) of the assisted serrations in the search space at a frequency range of 0–10,000 Hz with a tip-to-root ratio of 1.0, including (**a**) traditional serrations, (**b**) ogee-shaped serrations, (**c**) sinusoidal serrations, and (**d**) iron-shaped serrations.

**Figure 12:**
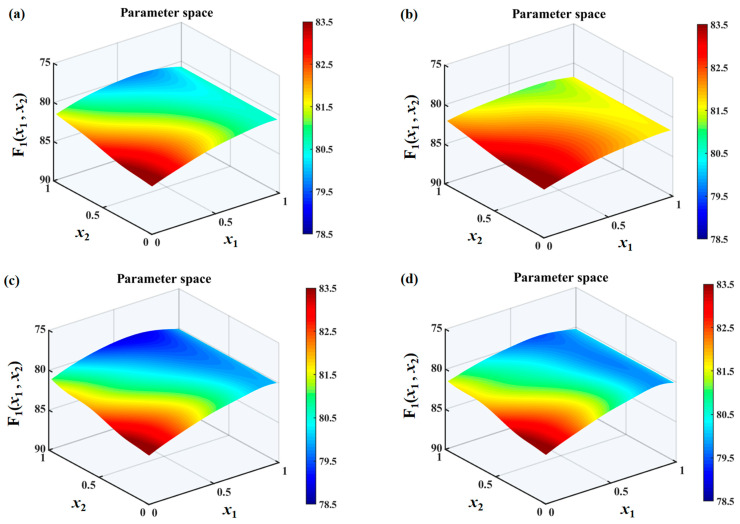
Sound pressure level distributions of a flat plate with double-wavelength serrations embedded and the optimization of the parameters of the wavelength coefficients (*x*_1_) for the main serrations and the amplitude coefficient (*x*_2_) for assisted serrations in the search space at a frequency range of 0–10,000 Hz with a tip-to-root ratio of 2.0, including (**a**) traditional serrations, (**b**) ogee-shaped serrations, (**c**) sinusoidal serrations, and (**d**) iron-shaped serrations.

The search spaces under different $\bar{h}$ values are shown in Figure 10, Figure 11 and Figure 12. It can be found that the overall sound pressure levels of flat plates with sinusoidal leading-edge serrations in the defined variable intervals are the smallest among the four kinds of serrated flat plates studied. With a continuous increase in $\bar{h}$, this advantage gradually becomes more significant. The noise-reducing ability of iron-shaped serrations is found to be comparable to that of sinusoidal serrations. The overall sound pressure levels of flat plates with the ogee-shaped serrations are the largest across the entire variable space, indicating that these serrations have a weaker ability to suppress noise emissions. When $\bar{h}$ is 1.0, a smaller sound pressure level can be easily obtained at a larger wavelength $x_{1}$. Therefore, the additional advantage of double-wavelength serrations is not significant at smaller $\bar{h}$ values. The smallest sound pressure level is distributed in approximately the middle of the wavelength at $\bar{h}=1.0$, and an additional noise reduction generated by double-wavelength serrations is gradually achieved. For serrated wavelength $\lambda_{2}$ embedded in the double wavelengths at different $\bar{h}$ values, the larger amplitude $x_{2}$ is conducive to noise reduction, which is in line with Howe’s early theory [34,35].

After optimization, the amplitude coefficient of the intermediate serrations is found to be one. At this value, the phase-cancellation interference between the root and tip of the serrations is enhanced, and this is consistent with the noise reduction trend shown in the previous research theory for single-wavelength serrations with larger $\bar{h}$ values. For the dominant wavelength $\lambda_{1}$ of the optimal double-wavelength serrations at $\bar{h}=2.0$ in this study, $\lambda_{1}=2\Lambda$ is satisfied. In a previous study, Chaitanya et al. proposed the concept of an optimal serration angle [$\theta ={tan}^{-1}(2h/\Lambda )$] for single-frequency serrations [36]. Here, it is confirmed that the wavelength at the optimal angle is about twice the turbulence integral length scale. This is consistent with the conclusion obtained in this study when $\bar{h}=2.0$. The commonality in the noise reduction mechanism between the single- and double-wavelength serrations is thus illustrated to a certain degree. Compared with single-wavelength serrations, double-wavelength serrations of different frequencies also contribute to additional noise-reducing capabilities due to phase differences.

**Figure 13:**
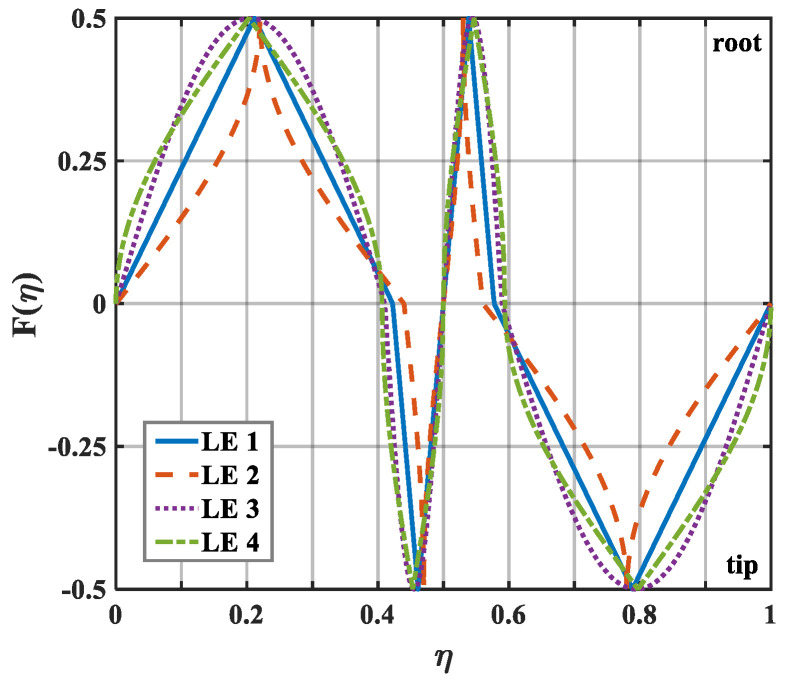
Schematic plot of double-wavelength serrations at a tip-to-root ratio of 0.5.

**Figure 14:**
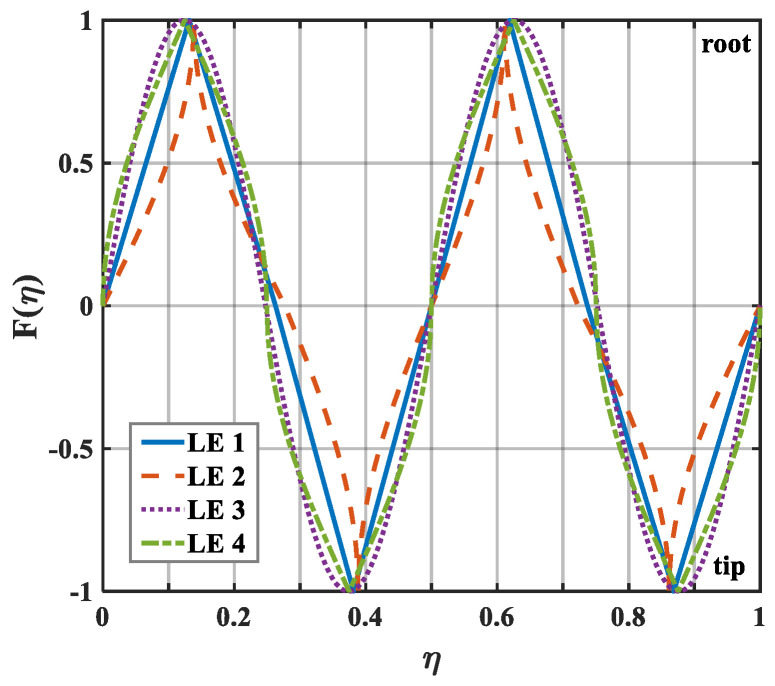
Schematic plot of double-wavelength serrations at a tip-to-root ratio of 1.0.

**Figure 15:**
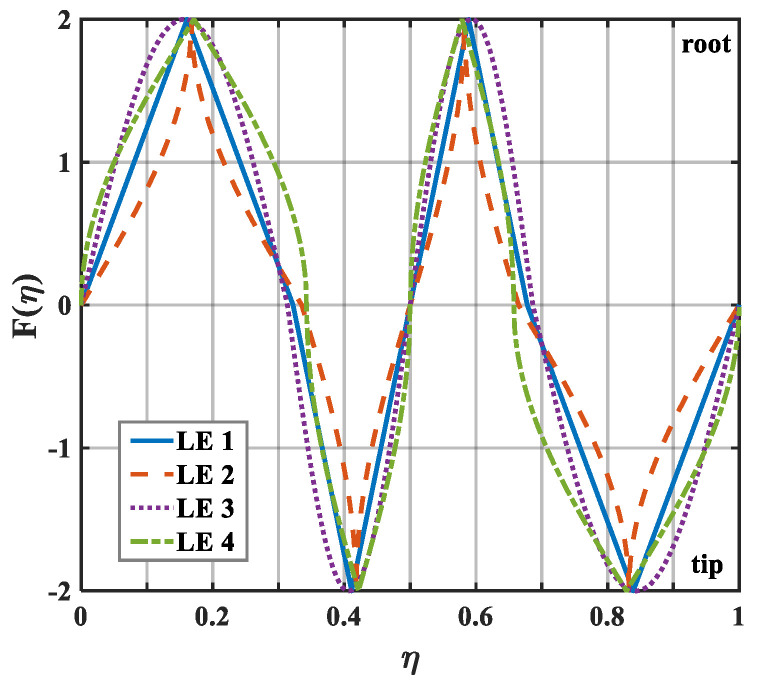
Schematic plot of double-wavelength serrations at a tip-to-root ratio of 2.0.

The geometric profiles with the minimum values of the overall sound pressure level at different tip-to-root ratios are illustrated in Figure 13, Figure 14 and Figure 15. Combined with Figure 1, the direction where F(η)>0 corresponds to the root of the serrations. On the contrary, the direction where F(η)<0 corresponds to the tip of the serrations. After optimization employing the whale optimization algorithm, it is found that the amplitude coefficients of the serrations are always one, which is similar to the conclusions reached by earlier researchers. This means that sharper auxiliary serrations are more likely to suppress noise emissions. The phase interference of the double-wavelength serrations also provides additional advantages for noise reduction.

**Figure 16:**
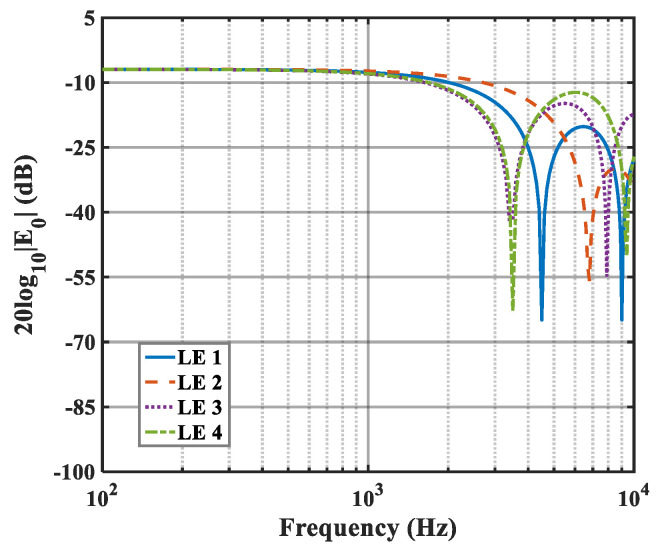
Plot of the decay rates of $\left|E_{0}\right|$ in relation to different optimal double-wavelength serrations at a tip-to-root ratio of 0.5.

**Figure 17:**
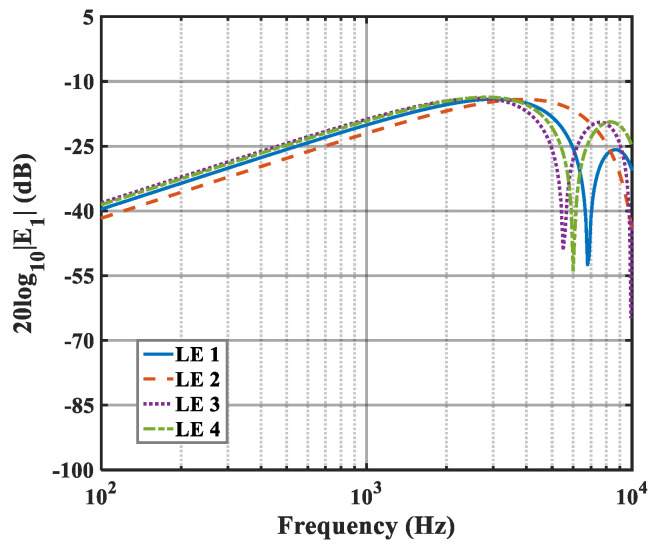
Plot of the decay rates of $\left|E_{1}\right|$ in relation to different optimal double-wavelength serrations at a tip-to-root ratio of 0.5.

**Figure 18:**
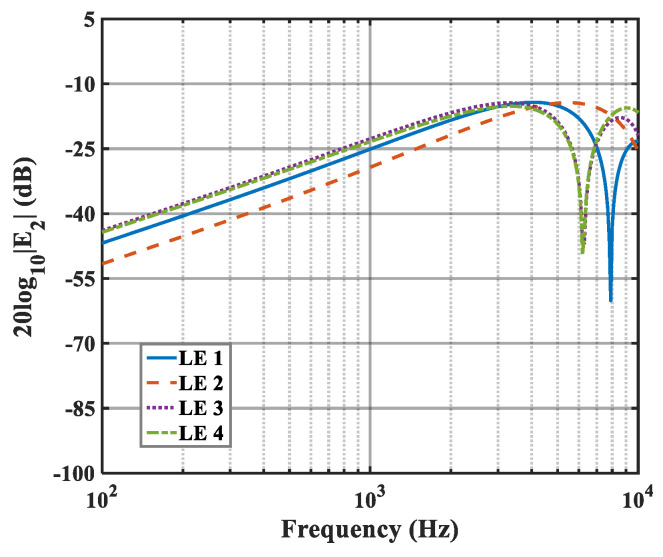
Plot of the decay rates of $\left|E_{2}\right|$ in relation to different optimal double-wavelength serrations at a tip-to-root ratio of 0.5.

**Figure 19:**
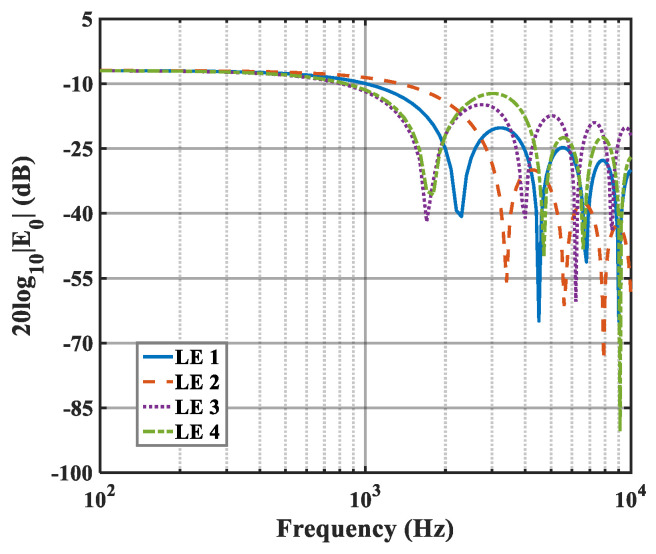
Plot of the decay rates of $\left|E_{0}\right|$ in relation to different optimal double-wavelength serrations at a tip-to-root ratio of 1.0.

**Figure 20:**
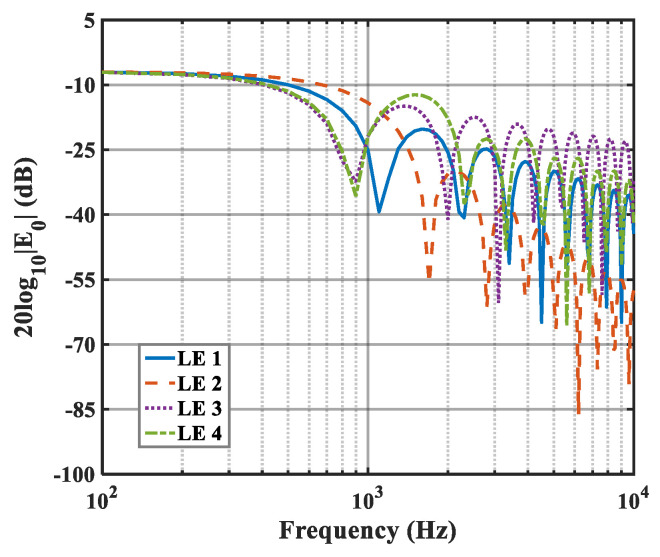
Plot of the decay rates of $\left|E_{0}\right|$ in relation to different optimal double-wavelength serrations at a tip-to-root ratio of 2.0.

The effects of zero- and higher-order modes of E*_n_* are also explored in Figure 16, Figure 17 and Figure 18. With increasing modal order, the dominant parts of E*_n_* for different serrations gradually tend toward higher frequencies. And, more modes need to be considered for higher frequencies. Since the higher-order modes contribute little to the sound pressure level at low frequencies, the calculation of higher-order modes is truncated to reduce the workload. Consequently, the zero-order dominant mode $E_{0}$, which is related to the number of degrees of freedom of the system, is now discussed. Distributions of $10{log}_{10}{\left|E_{0}\right|}^{2}$ for serrations with other tip-to-root ratios are shown in Figure 19 and Figure 20.

As $\bar{h}$ increases, noticeable noise-reducing effects can be observed over the entire frequency range. Especially in the high-frequency band, the noise reduction advantage of ogee serrations is significant. Nevertheless, the effectiveness of the leading-edge serrations is limited on account of the trailing-edge self-noise. Considering these results in combination with the subsequent spectra, it can be seen that the difference in the overall sound pressure level is mainly concentrated in the low-to-medium frequency range. In addition, the noise reduction trend for the function $10{log}_{10}{\left|E_{0}\right|}^{2}$ at lower frequencies is similar to the trends of the sound pressure level spectra. From this perspective, the function can be adopted as a trend estimate for the evaluation of noise reduction performance.

## 5. Results and Discussion

### 5.1. Analysis of the Radiation Integral Function ${\left|E_{n}\right|}^{2}$

The optimal serration arrangements of various shapes at different $\bar{h}$ values mainly depend on the shape function F(η). This function can take any value within the ranges of the variables. It is worth mentioning that the radiation integral related to ${\left|E_{n}\right|}^{2}$ with the smallest extremum is obtained by increasing the phase difference of the integrand function of Equation (2) [23]. It can be deduced that maximizing the amplitude of the serrations is conducive to optimizing the shape function. However, the aerodynamic performance of the flat plate can easily be affected by larger amplitudes, and so a serration amplitude within an appropriate range should be considered for actual operation. Howe previously studied a semi-infinite flat plate and proposed an interpolating prediction formula associated with the wavelength and amplitude of serrations at an intermediate frequency. It is found that when ωh/U≫1, the noise-reduction level is about $10{log}_{10}\left(1+{\left(4h/\lambda \right)}^{2}\right)/dB$ [34,35], and it is thus concluded that, under the condition of a fixed wavelength, a larger amplitude will contribute to greater noise suppression. Although the noise-reducing effect of the serrations is overestimated by this relation, the theoretical guidance has made great contributions to a certain degree.

**Figure 21:**
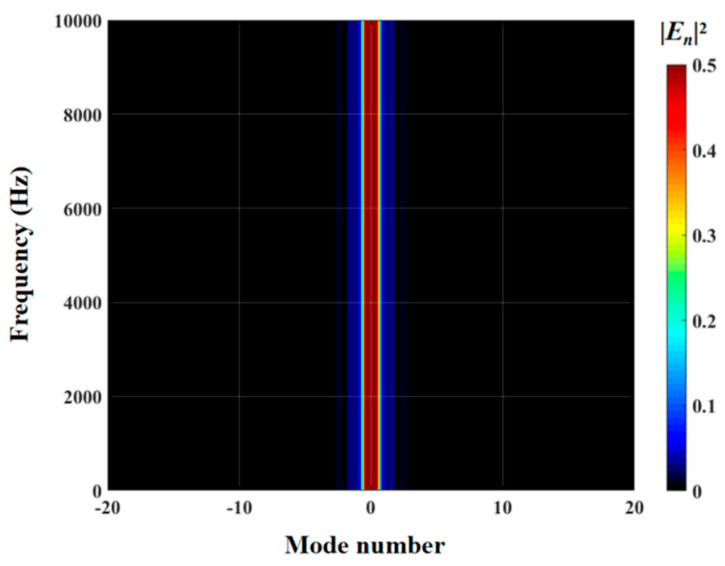
The contour of the radiation integral ${\left|E_{n}\right|}^{2}$ of a smooth leading edge.

**Figure 22:**
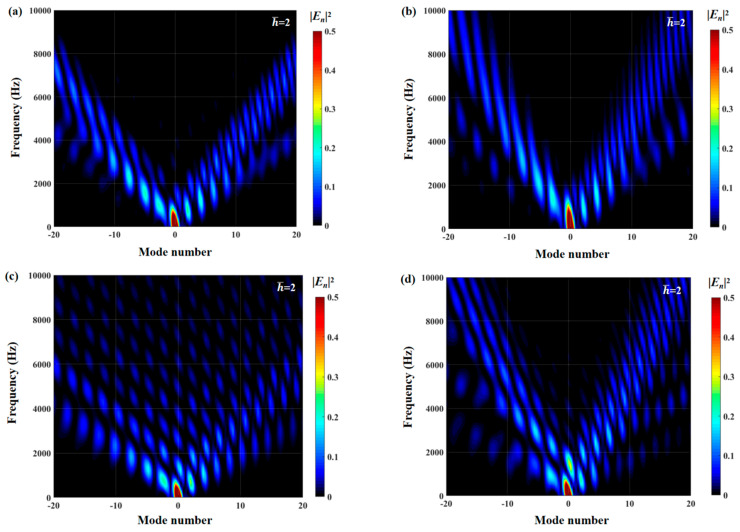
Contours of the radiation integral ${\left|E_{n}\right|}^{2}$ of optimal double-wavelength serrations based on the whale optimization algorithm at a tip-to-root ratio of 2, including (**a**) traditional serrations; (**b**) ogee-shaped serrations; (**c**) sinusoidal serrations; and (**d**) iron-shaped serrations.

As exhibited in Figure 21 and Figure 22, the maximum value of the radiation integral shows a gradual attenuation trend with increasing $\bar{h}$ values. The radiant integral of a smooth leading edge is only affected by the zero-order mode, which is related to the number of degrees of freedom of the system. What is different is that the serrations refactor the modal range of the airfoil with a smooth leading edge, which originally only functions in the dominant mode, and make the mode migrate to a wider range. The refactoring effect effectively attenuates the radiation integral intensity of the dominant mode. It is worth noting that the diffusion effect of double-wavelength sinusoidal serrations is more extensive than that of the other three serration types. In the low-frequency range, ogee-shaped serrations have a poor ability to decrease the radiation integral intensity of the dominant mode. Although the distribution range of the radiation integral intensity of sinusoidal serrations is minimal, this does not mean that sinusoidal serrations have the best noise-reducing level over the entire frequency band. For sinusoidal serrations, the radiation integral intensity of the dominant mode is greater than that of the other three serration types, while the ogee-shaped serrations present the opposite case. To be specific, it is difficult for any kind of serration to achieve full-band noise suppression in the dominant mode. It is thus necessary to suppress the corresponding wideband noise for specific cases.

### 5.2. Analysis of the Sound Pressure Level (SPL)

**Figure 23:**
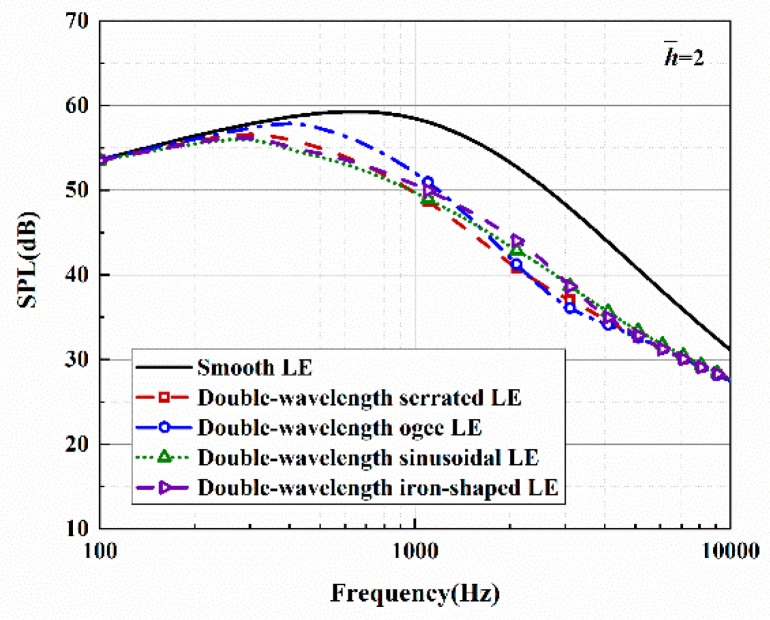
Sound pressure level spectra of optimal double-wavelength serrations at a tip-to-root ratio of 2.0.

**Figure 24:**
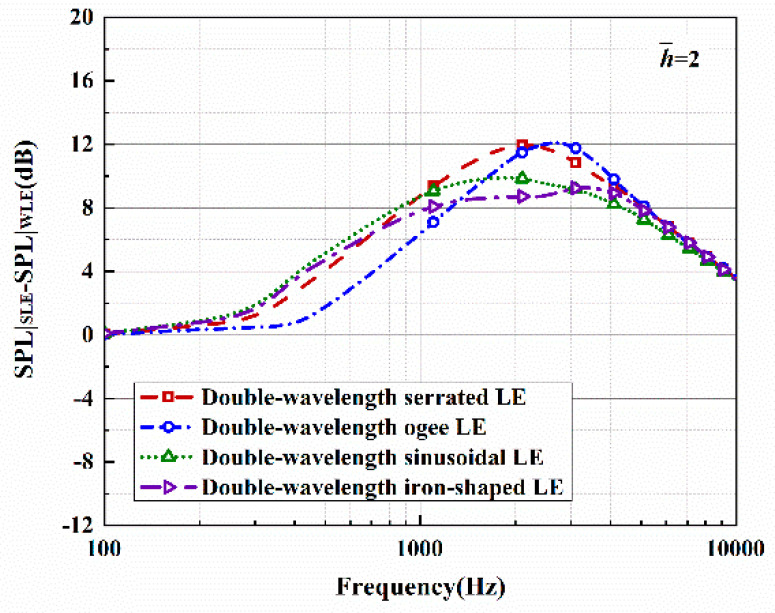
Plot of the sound reduction level of optimal double-wavelength serrations at a tip-to-root ratio of 2.0.

The sound pressure level and sound reduction levels in regard to the optimal double-wavelength serrations are depicted in Figure 23 and Figure 24. Here, the observation angle is set to 90°, and the Mach number is 0.17. The condition that $\left|k_{2}\right|$ must be less than $\left|k_{1}\right|$ also needs to be met. This operation not only satisfies the spanwise turbulent flows generated by the experimental equipment for a streamwise semi-infinite flat plate but also guarantees the assumption of finite sound waves. The sound pressure spectrum of a smooth leading edge is obtained by setting $\bar{h}=0.001$. The noise in the low- and mid-frequency rages gradually decreased as $\bar{h}$ is increased.

A maximum reduction level of 12 dB can be achieved at a specific frequency. The observed tendency of the sound pressure level to first increase and then decrease with increasing frequency agrees with the data extracted from experiments [22]. Low-frequency noise generally has the characteristics of strong penetration and high intensity, and it can thus be more harmful to people. For rotating machinery with low Reynolds numbers, the suppression of low-frequency noise often converts a noise reduction target aimed at the overall sound pressure level. Therefore, after solving the accuracy problem of the serrated structure in the high-frequency band, sound suppression in the mid-frequency and low-frequency bands is particularly significant.

### 5.3. Analysis of the Surface Pressure and Phase Distributions

The noise reduction mechanism of wavy leading edges is revealed by Kim et al. [37]. The source cut-off effect of the surface pressure is a result of the oblique geometric profile. The phase interference effect of the peak and hill centers of the serrations is conducive to sound suppression in the low-to-mid-frequency bands. Here, combined considerations of the surface pressure and phase with the above mechanisms are adopted to clarify the sound suppression.

The surface pressure is defined as

(18) $$p_{s}\left(x,y,0_{+}\right)=\sum_{n=-\infty }^{\infty }\frac{1}{2\pi \beta \sqrt{-{\bar{k}}_{1}-w_{n}}}\int_{-\infty }^{\infty }\frac{e^{-\frac{i\lambda x}{\beta }}E_{n}\left(\lambda \right)}{\sqrt{\lambda +w_{n}}}d\lambda e^{ik_{2}y+2n\pi iy}e^{-\frac{ik_{1}M^{2}x}{\beta^{2}}}$$

The surface pressure, which is closely related to far-field noise, is obtained for the independent variable hF(η). In contrast to a smooth airfoil, the modes of the serrated structure are reconstructed from the low modes to the high cut-off mode. While calculating the surface pressure, to make it possible to convert infinite wavenumbers into finite wavenumbers, the main contribution to the far-field sound pressure is the focus of our attention. In addition, single-frequency gusts are used to simplify the integration process, and the wavenumber $k_{2}$ manifested in the spanwise direction is fixed. These operations contribute to a clearer understanding of the phase interference and noise reduction mechanisms of the surface pressure redistribution generated by the serrated leading edges.

**Figure 25:**
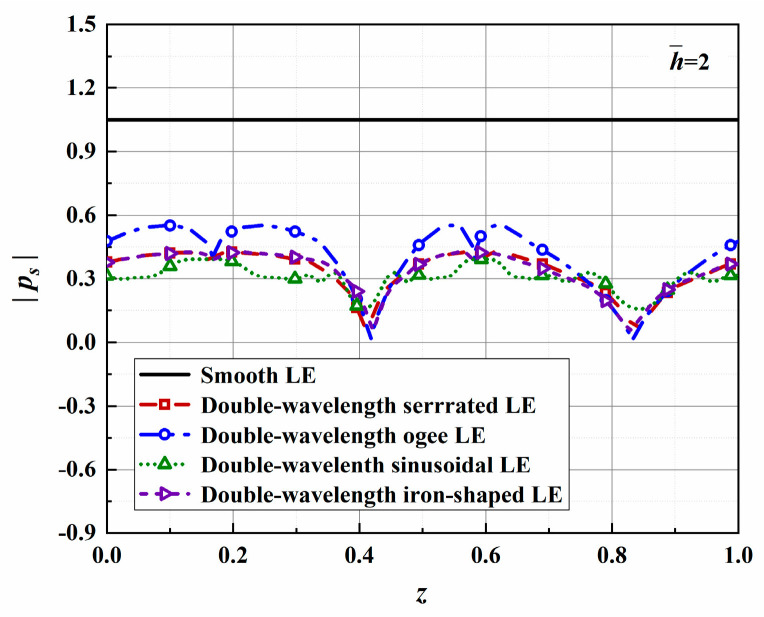
Plot of the absolute values of surface pressure promoting the transmission of acoustic waves with optimal double-wavelength serrations at a tip-to-root ratio of 2.

The surface pressures depicted in Figure 25 along with the leading edges in a uniform mean flow are calculated for $k_{2}=0$ at f=600 Hz. The sound source cut-off effect is confirmed while achieving different degrees of acoustic energy attenuation. Significantly, the distribution of surface pressure is closely related to the leading-edge geometry. The maximum reduction in the sound pressure level at the root reaches 44%. It is concluded that the sound source intensity of the serration roots is significantly decreased, and the source correlation is further weakened. The serrations rearrange the pressure distribution of the smooth flat plate to change the flow field, thereby reducing the intensity of acoustic energy radiation from the leading edge of the flat plate. It is once again confirmed that the analytical solution more quantitatively reflects the noise reduction mechanism speculated from computational fluid dynamic results.

**Figure 26:**
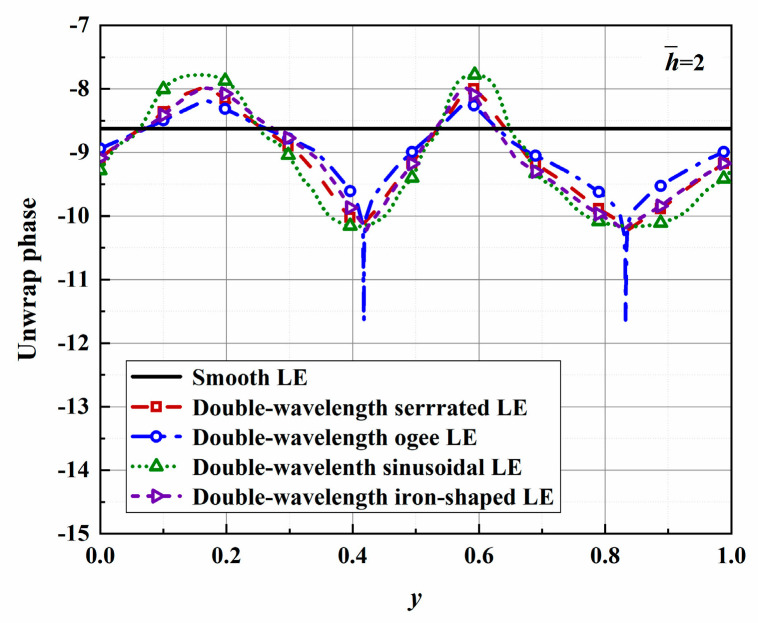
Plot of the phases of surface pressure along the spanwise leading edges for optimal double-wavelength serrations at a tip-to-root ratio of 2.

The phase distributions of four different serrations are shown in Figure 26. Consistent with the trends in the surface pressure distribution, the amplitudes of the phase differences between the tips and roots of the serrations also increase with increasing $\bar{h}$ values; the effectiveness of the phase interference noise reduction mechanism is thus verified. It is worth noting that for the ogee-type serrations, with increasing $\bar{h}$ values, the included angle formed by the root profile becomes increasingly sharp, and this means that a steep drop appears in the phase distribution of the root.

Considering these results in combination with the surface pressure distribution, it can be seen that the increase of the local phase difference will lead to a decrease in the local surface pressure, but it will not affect the overall distribution of the surface pressure. A phase difference of 2.39 reduces the surface pressure difference by an additional level of 0.24 at $\bar{h}=2.0$. This suggests that the local slits contribute to reducing the sound source intensity at the root. In practical applications, the existence of a leading edge of finite thickness cannot be avoided. Therefore, considering the specific design parameters to avoid the occurrence of Helmholtz resonance is necessary.

### 5.4. Directivity Performance of the Overall Sound Pressure Level

**Figure 27:**
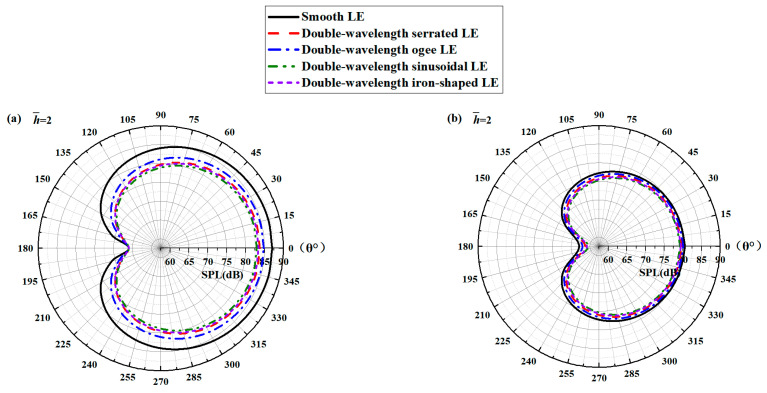

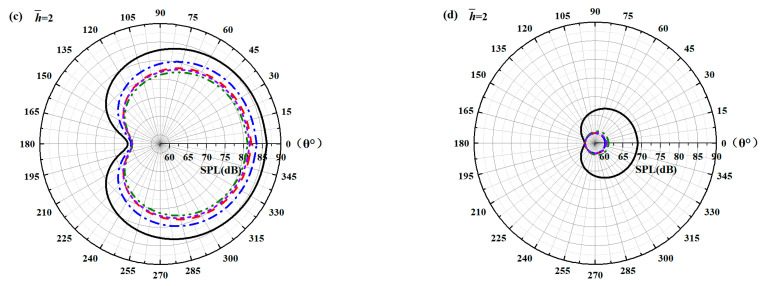
Polar plots of overall sound pressure levels integrated over different frequency ranges of (**a**) 0–10,000 Hz, (**b**) 0–500 Hz, (**c**) 500–5000 Hz, and (**d**) 5000–10,000 Hz at a tip-to-root ratio of 2.0.

According to the sound pressure level spectra, it can be observed that the noise at the specific frequency is well controlled in the low-frequency bands. The reduction in broadband noise integrated with different frequency bands has always been the focus and difficulty of people’s attention, which is closely related to the loudness of the A-weighted total sound pressure level received by the human ear. To elucidate the relationship between the noise reduction level of the double-wavelength serrations and the observer angle, the spatial directivity of the broadband noise divided according to different frequency bands is shown in Figure 27.

In addition, the application of serrations has an obvious effect on reducing the noise in all directions. The maximum reduction in the overall sound pressure level is 5.4 dB at $\bar{h}=2$. Compared with the other two serrations, sinusoidal and iron-shaped serrations improve the noise emissions in the low- and medium-frequency regions, whereas an undesirable slight increase in the sound pressure level in the high-frequency region is generated. The redistribution of the sound field energy is promoted to a certain degree.

### 5.5. Analysis of the Noise Reduction Mechanism of the Slits on Serrations

**Figure 28:**
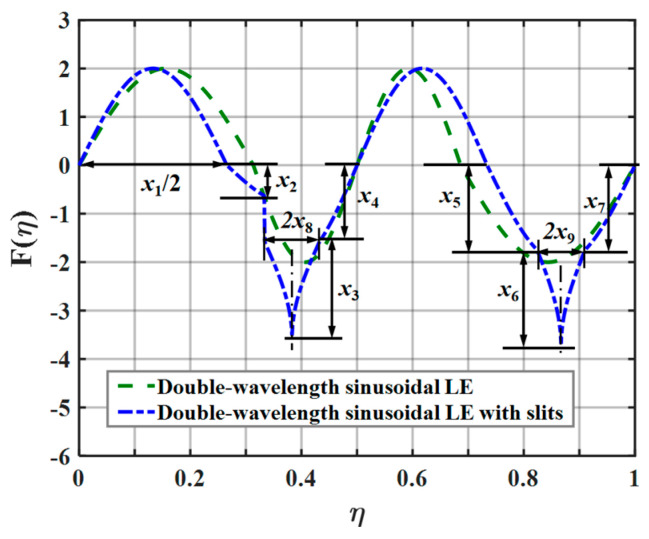
Sketch of the profile of optimal double-wavelength serrations obtained at a tip-to-root ratio of 2.0.

**Figure 29:**
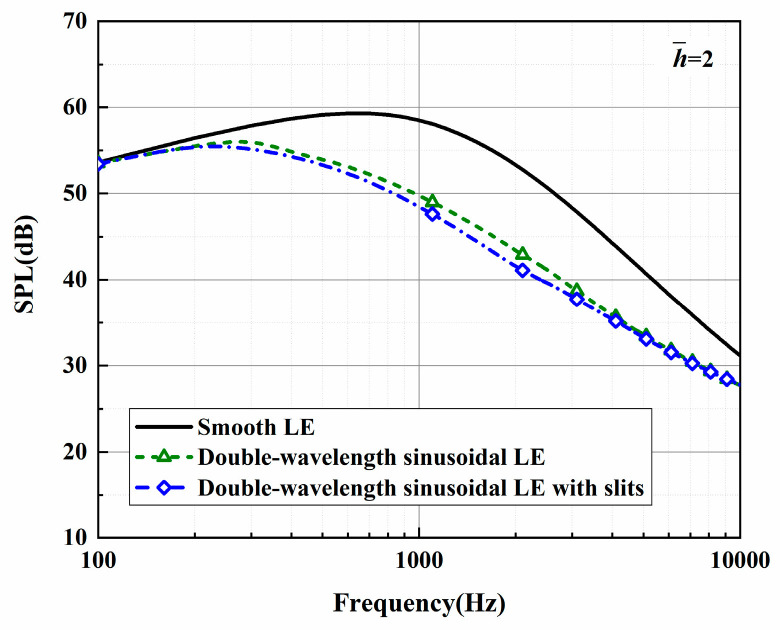
Sound pressure level spectra of optimal double-wavelength serrations with slits at a tip-to-root ratio of 2.0.

The above analysis is of significance for guiding the selection of serration profiles. The noise-reducing advantages of sinusoidal serrations across the whole spanwise distribution range and of ogee serrations in the local scope are thus combined in this study. All independent variables are controlled within a reasonable range. The whale optimization algorithm is employed to further optimize the geometric profile, and the serrations resulting from the corresponding optimal design parameters are shown in Figure 28. Here, the definition of the parameter $x_{1}$ is the same as in the previous settings, and the slits embedded in the roots of the sinusoidal serrations are in the form of a piecewise function: $x_{2}$ to $x_{7}$ are the amplitude coefficients of the sinusoidal and ogee-shaped serrations at the roots; and $x_{8}$ and $x_{9}$ are the slit widths on the left and right roots, respectively. The optimized parameters $x_{1}$ to $x_{9}$ of the serrations with slits are 0.5318, 0.4006, 1.0000, 1.0000, 1.0000, 1.0000, 1.0000, 0.0494, and 0.0430, respectively. When slits are introduced at the roots of the sinusoidal serrations, the optimal ratio of the wavelengths is redistributed. The asymmetric characteristic at both ends of the slits further contributes to a reduction in the sound pressure level. The sound pressure level spectra depicted in Figure 29 confirm that the implementation of slits weakens the noise emissions in the medium-frequency range, and an overall noise reduction effect of about 0.7 dB can be achieved.

**Figure 30:**
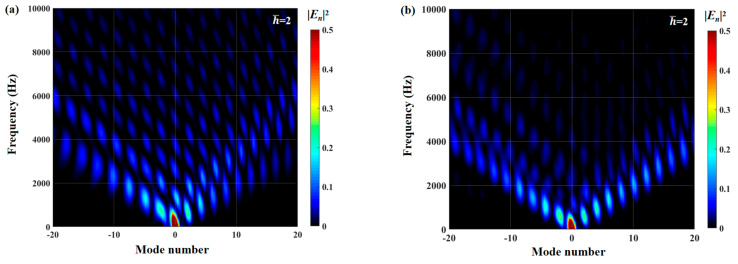
Contours of the radiation integral ${\left|E_{n}\right|}^{2}$ of optimal double-wavelength serrations with (**a**) sinusoidal profiles and (**b**) sinusoidal profiles embedded with slits obtained from the whale optimization algorithm at a tip-to-root ratio of 2.0.

**Figure 31:**
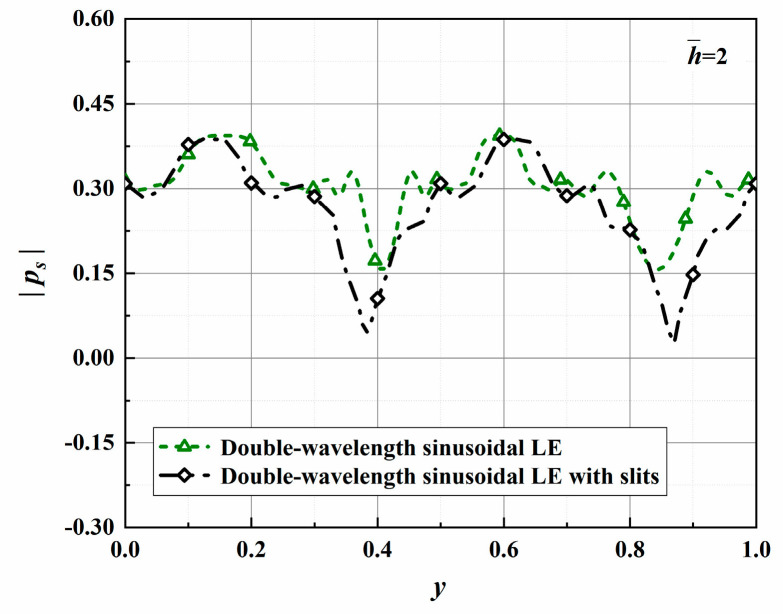
Comparison of the absolute values of surface pressure that promote the generation of acoustic waves for optimal double-wavelength serrations at a tip-to-root ratio of 2.0.

**Figure 32:**
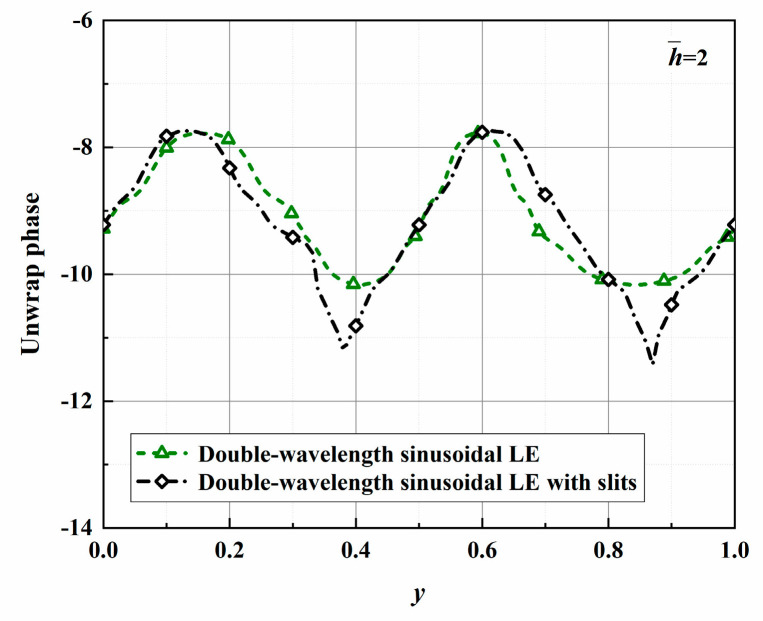
Comparison of the phases of surface pressure along the spanwise leading edges for optimal double-wavelength serrations at a tip-to-root ratio of 2.0.

The radiation integrals ${\left|E_{n}\right|}^{2}$ of the optimal double-wavelength sinusoidal serrations and the optimal double-wavelength sinusoidal serrations with slits are visualized in Figure 30. The most significant difference is manifested in the intensity of the integral radiation region of the dominant mode of the serrations with slits being decreased across a wide frequency range. The sound suppression of the sinusoidal serrations with slits is concentrated in the high-frequency range. Considering the distributions of the surface pressure and phase in Figure 31 and Figure 32, the minimum values at the root are further attenuated by up to 0.13 and 1.26, respectively. As a result of the asymmetric characteristics of the profile, both the surface pressure and phase distributions of the left adjacent profiles at the left root are decreased across a wide range along the leading edge. The coupled noise reduction mechanism caused by the scattering of the beveled edges and the root is thus revealed.

## 6. Conclusions

To reveal the noise reduction mechanisms of leading-edge serrated structures, a flat plate with the serrated leading-edge bio-inspired by bird wings is selected as the research object. The double-wavelength serrations are designed and adopted along the leading edges of a flat plate. The differences in the mathematical expressions of the serrations are illustrated within a cyclical range. With the purpose of further suppressing noise, the serrated structures are optimized based on the whale optimization algorithm. The main conclusions obtained in this study are as follows:

(1) The optimal amplitude coefficients of the dominant and auxiliary serrations are both one. The larger the amplitude of the serrations, the better the noise reduction level. This revealed some commonality between the noise reduction mechanisms of single- and double-wavelength serrations. When the tip-to-root ratio is large, the optimal dominant wavelength of double-wavelength serrations is closely related to the scale of twice the turbulence integral length.
(2) Among the four types of serrations with different tip-to-root ratios, serration profiles with convex functions contribute to reducing low-frequency noise, whereas serration profiles with concave functions are found to be conducive to decreasing high-frequency noise. However, because of the emission of trailing-edge self-noise, the advantage noise reduction of serrations with concave profiles in the high-frequency range is weakened under practical operating conditions.
(3) The wavelength difference of double-wavelength serrations promotes a further increase in the phase difference. The redistribution of energy from the lower cut-on modes to higher cut-off modes is promoted, and the relevant destructive interference effect of surface pressure is conducive to sound suppression. After the optimization and introduction of ogee-shaped slits based on sinusoidal serrations at the root, the radiation and source control are further improved. It is expected that these explorations will serve as a valuable reference for optimizing leading-edge serrations.
(4) Considering the inevitable impact of the self-noise generated by the trailing edge, the BPM model is used to predict the trailing-edge self-noise and improve the accuracy of the noise prediction of leading-edge serrated structures. The present method effectively improves the consistency between the predicted noise and experimental noise in the high-frequency region. However, there are still some unresolved questions in the current research, such as the influence of changes in the boundary-layer displacement thickness, which may lead to some deviations in the predicted results. In future work, we will carry out relevant studies to improve the accuracy of the prediction model.

## Data Availability

The data that support the findings of this study are available from the corresponding author upon reasonable request.
